# From inshore to offshore: distribution of microplastics in three Italian seawaters

**DOI:** 10.1007/s11356-022-23582-9

**Published:** 2022-10-21

**Authors:** Alice Sbrana, Tommaso Valente, Jessica Bianchi, Simone Franceschini, Raffaella Piermarini, Flavia Saccomandi, Andrea Giuseppe de Lucia, Andrea Camedda, Marco Matiddi, Cecilia Silvestri

**Affiliations:** 1 ISPRA, Italian National Institute for Environmental Protection and Research, Nekton Lab, Via di Castel Romano 100, 00144 Rome, RM Italy; 2 Department of Biology, PhD Program in Evolutionary Biology and Ecology, University of Rome Tor Vergata, Via della Ricerca Scientifica snc, 00133 Rome, Italy; 3 Department of Environmental Biology, Sapienza University of Rome, Piazzale Aldo Moro 5, 00185 Rome, Italy; 4 PhD Program in Ecology and Sustainable Management of Environmental Resources, Department of Ecology and Biology, University of Tuscia, Via S. Camillo de Lellis 44, 01100 Viterbo, VT Italy; 5 Hawaii Institute of Marine Biology, University of Hawaii, Kaneohe, HI USA; 6 IAS-CNR, Institute of Anthropic Impacts and Sustainability in Marine Environment–National Research Council Loc. Sa Mardini, 09170 Torregrande, OR Italy

**Keywords:** Microplastics, Sea surface, Mediterranean Sea, Monitoring, Coastal distance, Distribution

## Abstract

A comprehensive understanding of the concentration of microplastics (MPs) in seawaters is essential to implement monitoring programs and understand the impacts on ecosystems, as required by the European legislation to protect the marine environment. In this context, the purpose of this study is to investigate the composition, quantity, and spatial distribution of microplastics from coastal to offshore areas in three Italian seawaters. In addition, the distribution of microplastics between surface and subsurface water layers was analyzed in order to better understand the dynamics of MPs in the upper layers of the water column. A total number of 6069 MPs (mean total concentration of 0.029 microplastics · m^−2^) were found to be heterogeneous in type, shape, and color. In general, MPs concentrations decrease with coastal distance, except when environmental forcings are predominant (such as sea currents). Moreover, the amount of surface MPs was almost four times that of subsurface microplastics, which consisted mostly of fibers. In light of these results, it becomes clear how critical it is to plan remediation actions and programs to minimize microplastic accumulations in the sea.

## Introduction

The production and the use of plastic materials increased exponentially during the twentieth century (Barnes et al. 2009). The plastic development process has led to inevitable consequences, with repercussions on waste management and negative impacts on terrestrial and aquatic ecosystems (Lebreton et al. 2019; Thompson et al. 2009).

Globally, in 2010, the accumulation of macro-and microplastics in the ocean surface layer was 4210 tons and 1130 tons per year, respectively (Koelmans et al. 2017). Despite the rapid increase in plastic emissions, macroplastic and microplastic abundances have continued to rise steadily until now, indicating that concentrations in the ocean surface layer have not reached a steady state (Koelmans et al. 2017). The majority of the plastics entering the marine environment have degraded into micro- and nanoplastics, which makes the prediction of their presence more difficult (Lebreton et al. 2019).

Microplastics (MPs) are widespread in seas and oceans, mostly found on the surface but also in the water column and marine sediments (Ryan et al. 2009). The spatial distribution of this pollutant is influenced by multiple interacting factors (Franceschini et al. 2019). In particular, floating microplastics are carried by seawater movements and their distribution will reflect the surface and winds circulation (Iwasaki et al. 2017; Reisser et al. 2015). In coastal areas, multiple anthropogenic factors can affect their accumulation and dispersal (Suaria and Aliani 2014; Thiel et al. 2013). Proximity to big cities and anthropogenic activities (e.g., coastal tourism, recreational boating, agriculture, ports, industrial activities, fishing, aquaculture) can significantly contribute to the amount of marine litter in the marine environments (Araújo and Costa, 2007; Jambeck et al. 2015; Rech et al. 2014; Thiel et al. 2013; Hanke et al. 2013; Lusher et al. 2017).

The abundance, persistence, and ubiquity of MPs represent a crucial factor in environmental pollution and a serious threat to marine organisms. Indeed, the smaller size of MPs increases the probability of plastic ingestion by individuals (Auta et al. 2017; Kühn et al. 2015; Tsangaris et al. 2020; UNEP/MAP SPA/RAC 2018; Valente et al. 2019; Werner et al. 2016).

In 2008, the European Union approved the Marine Strategy Framework Directive (MSFD, 2008/56/EC) to protect more effectively the marine environment across Europe. The overall achievement of the MSFD is the Good Environmental Status (GES). One of the major objectives is to reduce the loss of marine biodiversity and to monitor the concentration of pollutants in marine environments, and their impact on marine biota. In particular, the Marine Litter survey (D10 Marine Litter) is aimed at protecting the marine environment against harm caused by litter. Despite the lack of a baseline for microlitter values in European seawaters, several scientific studies have found that considerable amounts of microlitter are present in seawaters, which are directly related to the amount of litter present in terrestrial and riverine environments. The Directive requires Member States to define GES at the level of the region or subregion, plan to set threshold values, perform regular assessments, and implement programs of measures (2017/848/EU). The assessment of the current environmental status requires a comparison between a reference (expected/usual/normal) state and an impacted one (Werner et al. 2020). Thus, it is necessary to define the reference values for indicators against which the actual or potentially changed situation can be compared. Nowadays, the definition of the threshold value is computed only for beach litter (Van Loon et al. 2020). Regarding microlitter, there is a lack of coherence within the same marine region or subregions and harmonized sampling and laboratory methods need to be agreed (Werner et al. 2020). Method of sampling and laboratory analysis conducted by different countries are often inconsistent and there is a fundamental absence of comparability among data (Covernton et al. 2019; Hermsen et al. 2018). Furthermore, inconsistency can be seen in the absence of a definition of microplastics size. In actuality, whilst the upper limits are fixed (5 mm, Arthur et al. 2009), the lower limits remain unclear. It is therefore important to increase marine MPs concentration data and to develop standardized sampling protocols to be comparable across studies and regions.

In this study, we investigated the composition (polymeric types, shapes, and colors), amount, and spatial distribution of MPs particles from coastal to offshore areas in three Italian seawaters. Furthermore, we examined differences in microplastic composition between surface and subsurface waters, in order to understand the dynamics of microplastics in the upper layers of the water column. The results of this study provide a better understanding of MPs pollution, both in a spatial and vertical context, and contributes to increasing data and assessing potential implications for future MSFD monitoring programs.

## Methods

### Sampling activities

The study involved sampling in coastal and offshore areas of the three Italian subregions, defined by the Italian national microlitter monitoring protocol for the MSFD, and developed by the Ministry of the Ecological Transition (MiTE), the Italian National Institute for Environmental Protection and Research (ISPRA), and the Regional Environmental Protection Agencies (ARPAs). As a result, samples were collected during 2019 and 2020 from the following areas:

- The Adriatic Sea (MAD);
- The Western Mediterranean Sea (MWE);
- The Ionian Sea and the Central Mediterranean Sea (MIC).

**Fig. 1:**
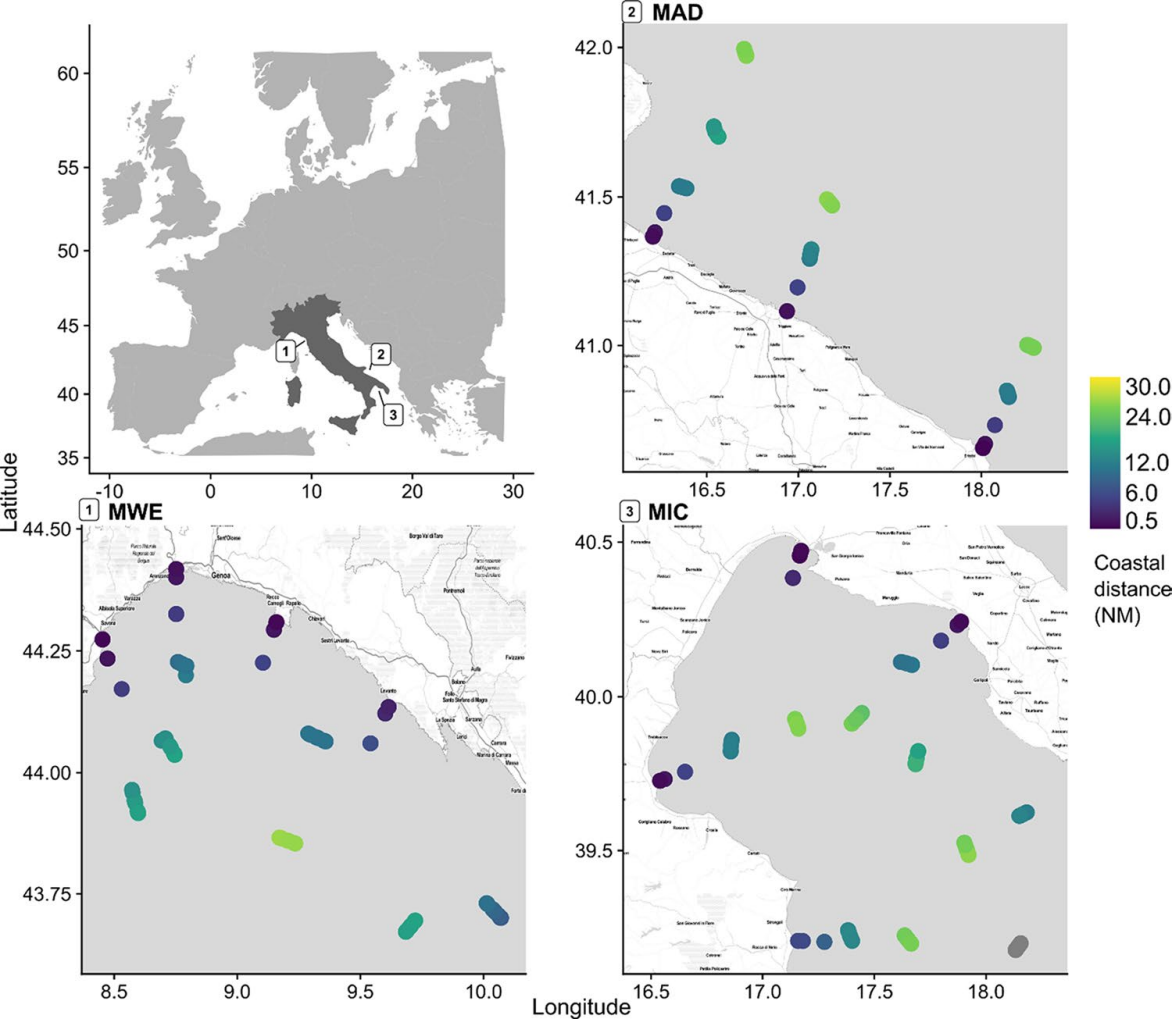
Seawater samples collected from the three Italian MSFD sub-regions: MWE, Western Mediterranean Sea (including the Ligurian and Tyrrhenian Seas); MAD, Adriatic Sea; and MIC, the Ionian Sea, and Central Mediterranean Sea. Colors indicate the distance from the coast in nautical miles (NM)

Stations were selected within 0.5 to 6 nautical miles (~ 0.9 to 11 km) off the coast, based on several environmental characteristics and anthropogenic factors (i.e., upwelling and downwelling areas, storage areas for local hydrodynamic conditions, distance from direct input sources such as river mouths, and distance from port facilities or relevant urban settlements). Offshore stations were located at 12 and 24 nautical miles (~ 22 to 44 km) off the coast, following the tracks of coastal ones (Fig. 1). Samples of coastal waters were collected by the 3 ARPAs of the Italian administrative regions (Liguria, Calabria, and Puglia) between spring and autumn. During the summer, ISPRA completed the offshore sampling.

Several studies estimated the vertical distribution of MPs in the water column depending on environmental variables (Kukulka et al. 2012; Kooi et al. 2016), instrumental mesh size (Green et al. 2018; Karlsson et al. 2020; Viršek et al. 2016; Zheng et al. 2021) or particle densities (Choy et al. 2019; Lenaker et al. 2019), considering different methods to sample seawater MPs. The most common approach is the surface net tow, using a manta trawl, to collect floating MPs, while for sampling in subsurface waters, a plankton net is typically used (Cutroneo et al. 2020). Samples from the sea surface layer were collected by using a manta net (mouth 50 × 25 cm, net: mesh size 330 µm). Manta nets were trawled for 20 min in rectilinear transects with a speed of 1–2 knots (~ 1.8 to 3.7 km/h) in the opposite direction from the wind and surface current. GPS coordinates were recorded at the beginning and end of each trawl. In each station, three consecutive withdrawals were obtained.

The surface area of surveyed water (S) was calculated by applying the following formula:

$$S=D\times W$$

Where *D* is the distance covered by the net, and *W* is the width of the net (0.5 m).

**Fig. 2:**
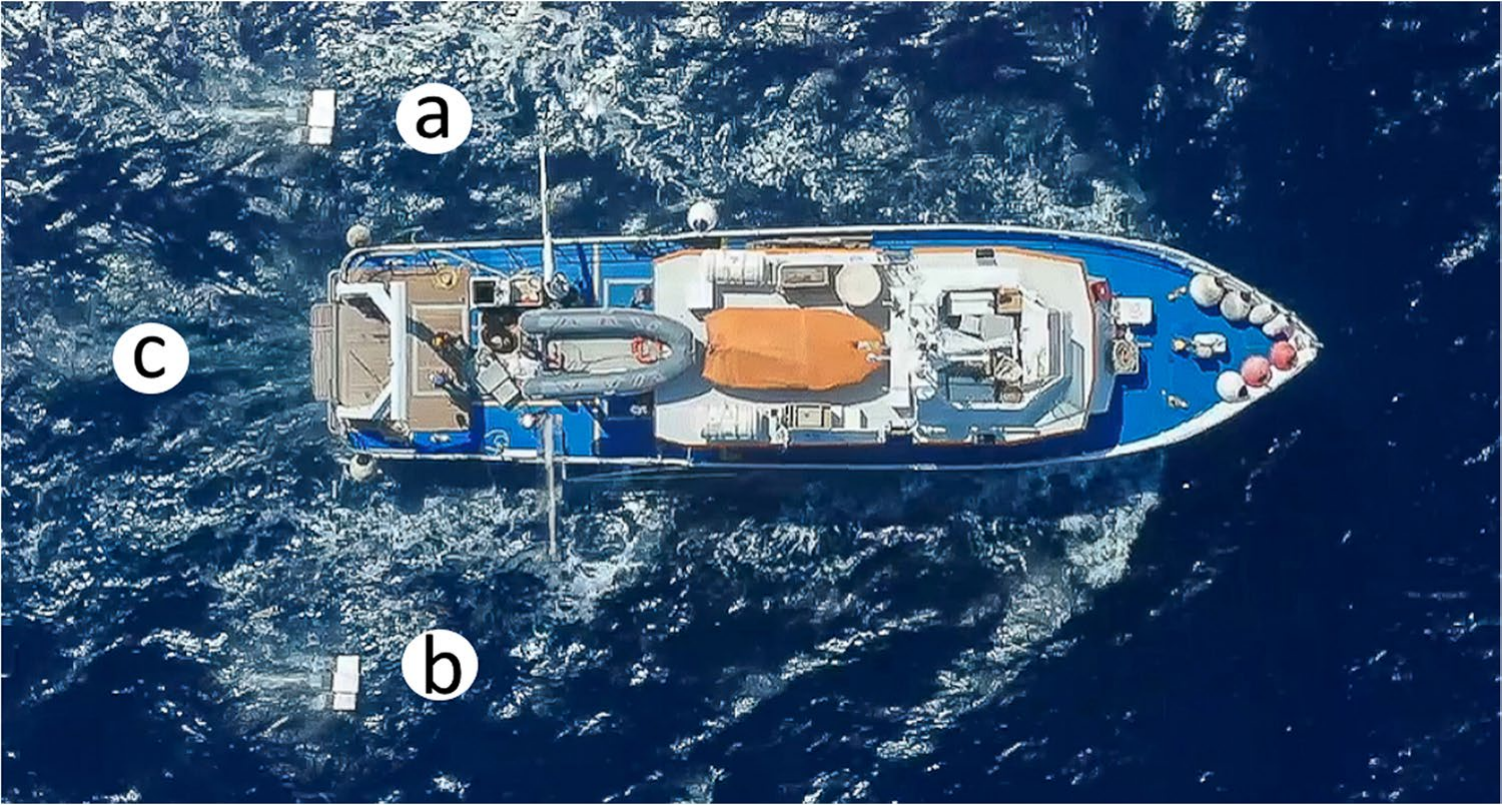
Sampling method for seawater microplastics using simultaneously two manta nets, one to the left (port side; **a**), one to the right (starboard; **b**), and a plankton net (**c**) at a depth of 10–20 m

During the same transect of the offshore stations, two twin manta nets, one on the port side (Fig. 2a) and one on the starboard side (Fig. 2b) of the stern ship, were simultaneously trawled. Additionally, subsurface samples were collected in the same sites at depths of 10–20 m using a plankton net wp2 (diameter 50 cm, mesh size 330 µm; Fig. 2c). The collected samples were treated separately to detect any differences in the distribution and composition of microparticles.

Upon returning to the ship, the nets were rinsed with seawater from the part near its mouth to the collection sock. To reduce the volume of samples, larger pieces of biological material, such as leaves, bugs, large algae, or wood, were carefully removed from the samples using metal tweezers and rinsed on a metal sieve (mesh size < 330 μm). To prevent the overestimation of particle quantification (i.e., fragmentation of the larger particles into MPs), a similar method was used for the removal of macroplastics particles (> 5 mm) from the main sample. The collected materials were placed in a 500 ml glass bottle for further analysis and stored in refrigerators at 4 °C or room temperature, protected from light and heat.

### Laboratory analyses

In the laboratory, samples were treated with 15% H_2_O_2_ to digest organic substances. Hydrogen peroxide solution was added at a 1:1 volume, sample: solution ratio, and stored 5 days at room temperature. Afterward, samples were vacuum filtered through a Whatman GF/D™ filter (47 mm in diameter, pore size 2.7 µm).

Items were visually sorted and identified under a stereomicroscope (Carl Zeiss Stemi 2000-C microscope; zoom range: × 0.65– × 5.0; total magnification range × 6.5– × 50). Microplastics (referred as particles smaller than 5 mm and larger than 330 μm, due to the lower limit mesh size of the sampler) were identified based on modified Norén (2007) protocol: (i) the resistance of the particles to the contact with tweezers; (ii) the absence of cell structures; (iii) either typical skewed shapes and crooked edges or uniform thickness; (iv) distinctive colors. Furthermore, we considered plastic items showing a dark sticky mark when touched with a hot needle (Hermsen et al. 2018). Polymer characterization was performed by taking a 10% sub-sample of offshore plastic particles (total items = 300), as specified by the Guidance on Monitoring of Marine Litter in European Seas (Hanke et al. 2013), and using Nicolet iS10 Fourier Transform Infrared Spectroscopy with Attenuated Total Reflection (ATR-FTIR) (Thermo Fisher Scientific, Madison, WI, USA).

All the collected particles were sub-divided into 6 shape categories (fiber, filament, foam, sheet, fragment, pellet), according to Matiddi et al. (2021).

To avoid secondary contamination, a Tyvek® protective suit was used during all laboratory phases, and samples were processed under a laminar flow cabinet. Filters were stored in covered glass Petri dishes and all laboratory instruments and tools were washed with ultra-pure water and checked under a stereomicroscope, to prevent cross-contamination. Procedural blanks were used in all steps (digestion, filtration, and identification) for each batch of processed samples (about 10 samples). Since the average microplastic contamination was minimal (average plastic particles in blank sample < 3), we did not subtract contamination from the field sample results.

### Data analyses

Microplastic concentration was expressed as the number of particles per surveyed area (items· m^−2^). Firstly, a data exploration was performed to detect outliers, assess the collinearity of the explanatory variables, and relationships between the response variable and the explanatory ones (Zuur et al. 2009). The Shapiro-Wilks test was used to test for normality in the data. Accordingly, the data were not normally distributed (*p* > 0.05). Therefore, the log transformation was used for statistical analysis.

On surface samples collected with the manta net, generalized linear models (GLMs) were used to analyze the interaction between environmental variables (distance from the coast, depth, wind speed) and MPs log-transformed concentrations. Model selections were based on the information-theoretic approach (Burnham and Anderson 2007), by comparing models AICs (Akaike’s Information Criterion; Akaike 1974). A significant difference was attributed where *p* < 0.05.

A nonmetric multidimensional scaling (nMDS) plot was used to examine the shape of microplastics (i.e., by particle typology—fiber, filament, foam, sheet, fragment, pellet) among surface—subsurface seawaters. The plot considered both the number of microplastics as well as the shape of particles within each sample. It grouped samples with similar patterns based on both amount and typology. For nMDS plots two-dimensional ordinations using “metaMDS” and Bray–Curtis dissimilarity were made.

Statistical analyses were conducted with R 4.0.4 (R Core Team 2021), using ggplot2 (Wickham 2016), ggmap (Kahle and Wickham 2013), and vegan (Oksanen 2020) packages.

## Results

During the surveys, 249 water samples were collected (about 80 samples for each subregion). FT-IR identification confirmed that all the isolated particles were plastic polymers: 72% polyethylene (PE), 24% polypropylene (PP), and 4% polystyrene (PS). In each subregion, the majority of samples contained plastic-like microparticles, whereas only 31 samples did not contain MPs. A total number of 6069 microplastics were detected, with a mean total concentration of 0.029 microplastics · m^−2^.

**Fig. 3:**
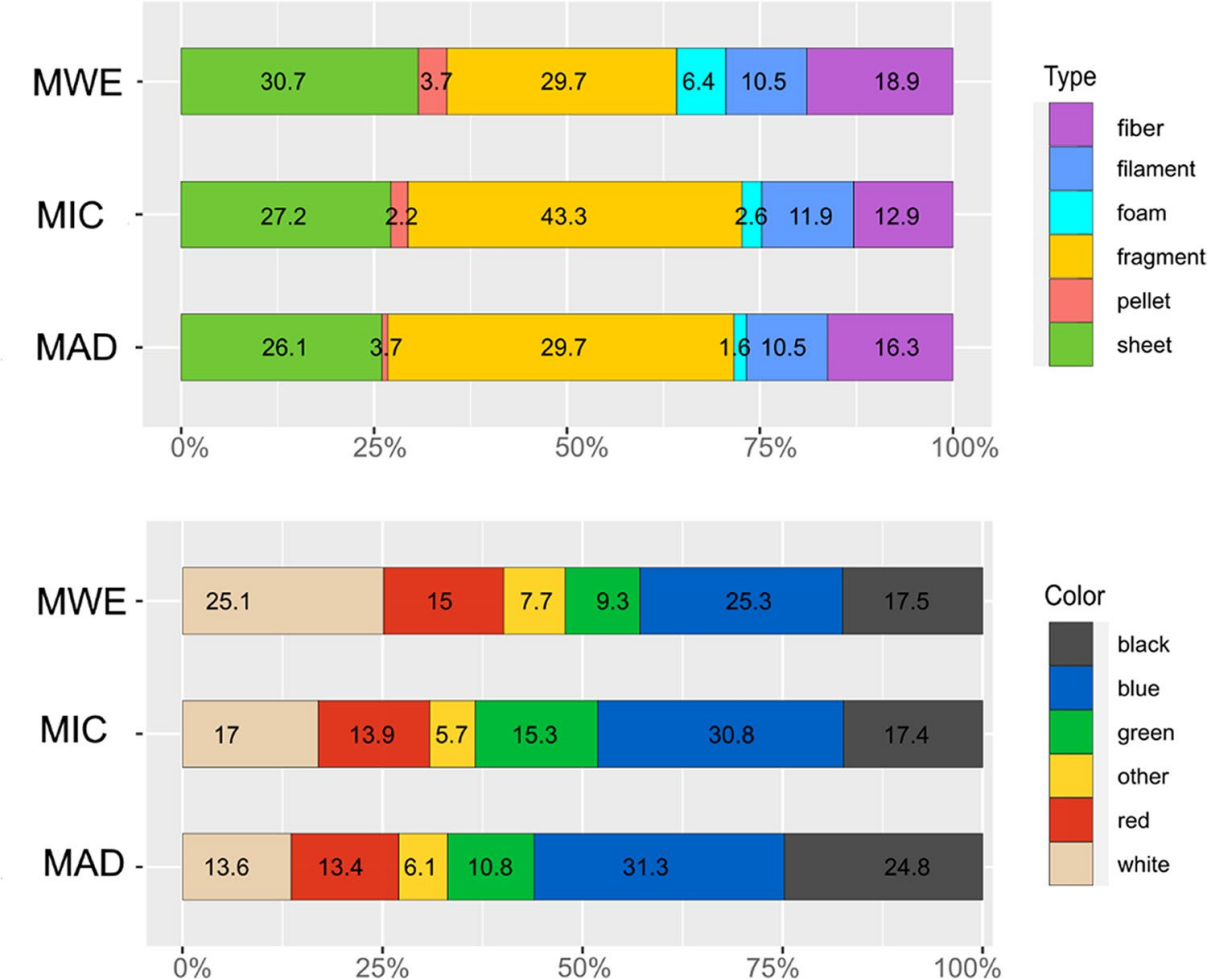
Shape categories and colors of microplastics collected in seawater in areas of the three MSFD subregions: MWE, the Western Mediterranean Sea (including the Ligurian Sea, and the Tyrrhenian Sea); MAD, the Adriatic Sea; and MIC, the Ionian Sea, and Central Mediterranean Sea

Overall, particles varied by shape and color (Fig. 3). Fragments were the predominant types (39%) followed by sheet (28%), fiber (17%), filament (11%), foam (3%), and pellet (2%). We found a great variety of colors, ranging from dominant blue items (29%) to black (20%), white (18%), red (14%), green (12%), and others (7%).

**Fig. 4:**
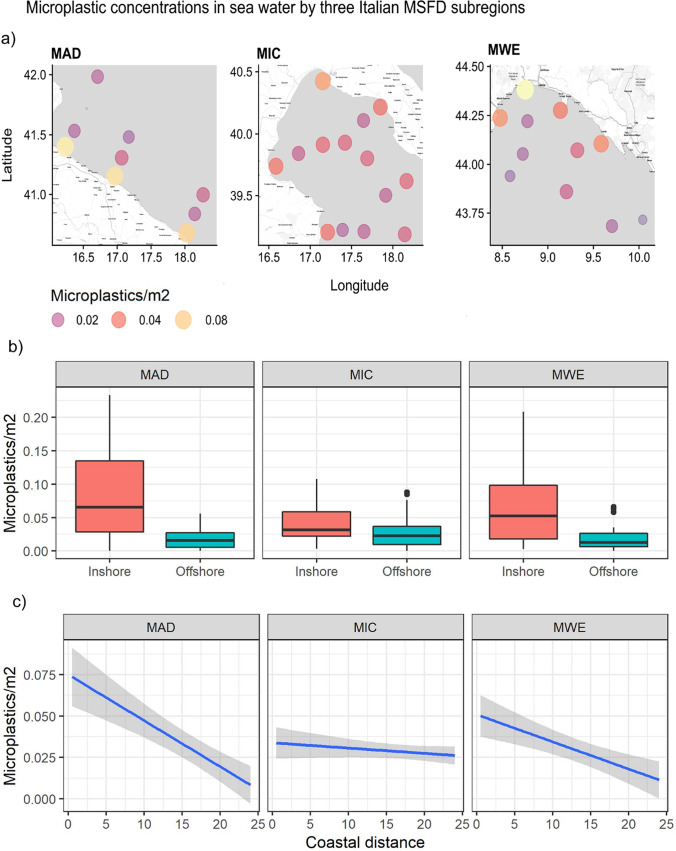
Seawater microplastics concentration (**a**), differences between coastal distance (**b**), and the effects of coastal distance, resulting from GLM model, as a predictor for MPs concentration (**c**). Confidence intervals (95%) around the response curve are shown in grey. Areas of the three Italian MSFD subregions: MWE, the Western Mediterranean Sea (including the Ligurian Sea and the Tyrrhenian Sea); MAD, the Adriatic Sea; and MIC, the Ionian Sea, and Central Mediterranean Sea. Offshore waters range from 12 to 24 NM; inshore waters range from 0.5 to 6 NM

Seawater concentration of microplastics did not show significant differences among subregions (Fig. 4a, Table 1). The coastal sites (inshore) had the highest concentration of microplastics (MPs·m^−2^ average = 0.062) compared to offshore waters (MPs·m^−2^ average = 0.020) (Fig. 4b).

**Table 1:** Concentration of microplastics· m^−2^ in seawater (including the 10th/90th percentile, median, and mean) by coastal distance (inshore–offshore) in three Italian MSFD subregions: MWE, the Western Mediterranean Sea (including the Ligurian Sea and the Tyrrhenian Sea); MAD, the Adriatic Sea; and MIC, the Ionian Sea, and Central Mediterranean Sea

Coastal distance	Sea	10th percentile	Median	90th percentile	Mean
*Inshore (*< *12 NM)*	*MAD*	*0.014*	*0.065*	*0.202*	*0.091*
	*MIC*	*0.011*	*0.031*	*0.097*	*0.041*
	*MWE*	*0.012*	*0.052*	*0.111*	*0.062*
*Offshore (*> *12 NM)*	*MAD*	*0.002*	*0.016*	*0.035*	*0.017*
	*MIC*	*0.002*	*0.022*	*0.048*	*0.025*
	*MWE*	*0.002*	*0.012*	*0.033*	*0.017*

The best-fit GLM model, with the lowest AIC (Table 2), included only distance from the coast as a predictor of MPs concentration. The summary of the model showed a significant decrease in microplastics with increasing distance from the coast in samples from the Adriatic Sea (Fig. 4c, MAD) and from the Ligurian Sea (Fig. 4c, MWE), while it is not detected in samples from the Ionian Sea (Fig. 4c, MIC).

**Table 2:** Summary of the results from the best-fit generalized linear model (GLM) (including only coastal distance) in areas of the three Italian MSFD subregions: MWE, the Western Mediterranean Sea (including the Ligurian Sea and the Tyrrhenian Sea); MAD, the Adriatic Sea; and MIC, the Ionian Sea, and Central Mediterranean Sea

Sea		Estimate	Std. error	Z value	P value
MAD	*log (MP·m*^*−2*^*)*	0.4318152	0.0164854	26.194	***
	*Dist. Coast*	− 0.0039383	0.0008703	− 4.525	***
MIC	*log (MP·m*^*−2*^*)*	0.3841899	0.0132278	29.044	***
	*Dist. Coast*	− 0.0008016	0.0006869	− 1.167	–
MWE	*log (MP·m*^*−2*^*)*	0.3928887	0.0131887	29.790	***
	*Dist. Coast*	0.0022711	0.0007992	− 2.842	**

The *estimate* represents the average change in the response variable that results from a unit increase in each predictor variable. *Standard errors* indicate the degree of uncertainty in the estimate. *The z* value is obtained by dividing the estimate by the standard error. *P value* indicates how well each predictor variable predicts the value of the response variable in a model. Significance codes: *p < 0.05; **p < 0.01; ***p < 0.001

**Fig. 5:**
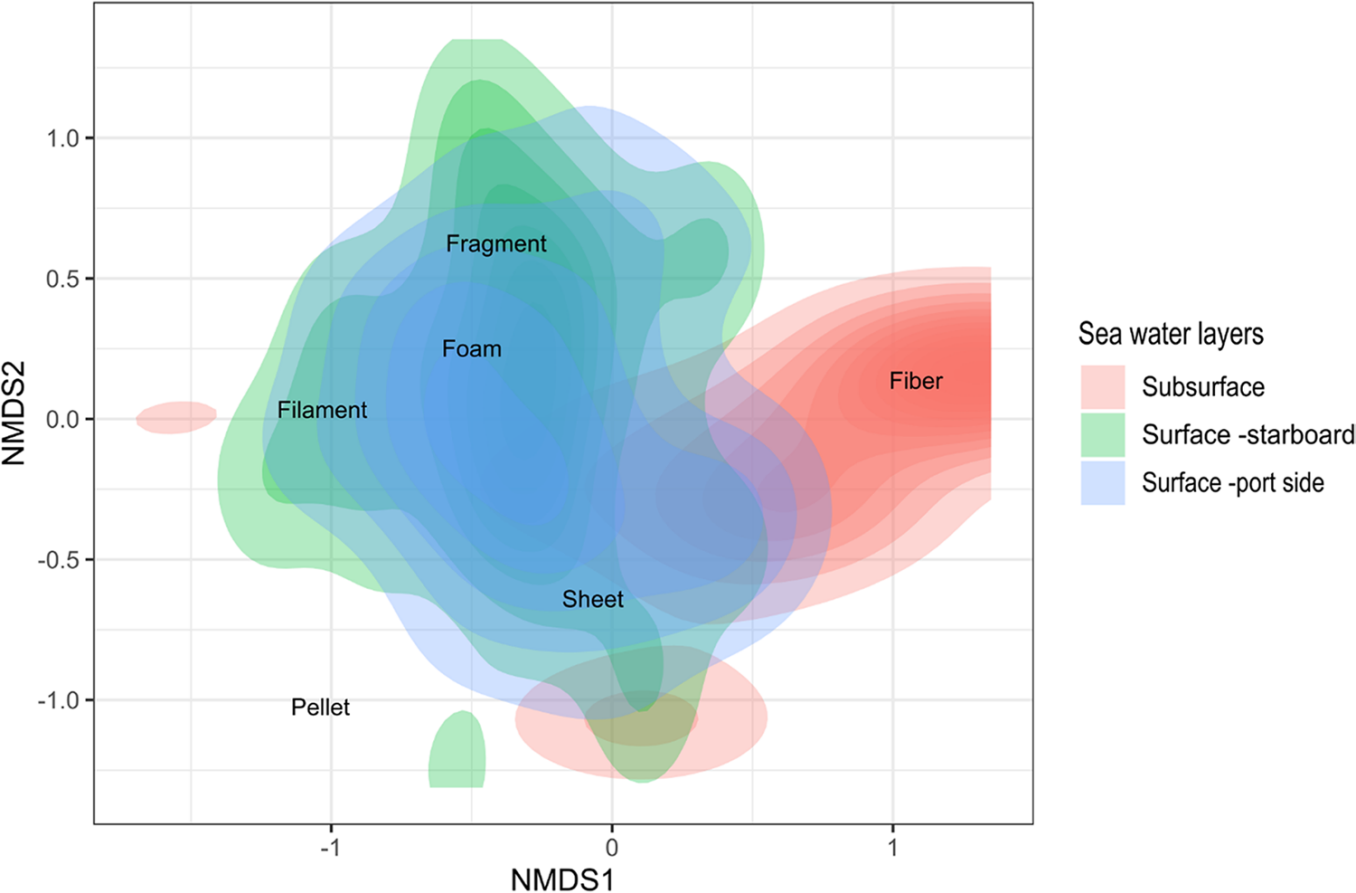
A nonmetric multidimensional scaling plot created using the number of microplastics typologies within each seawater layer (starboard–port side surface, and subsurface waters) in areas of the three Italian MSFD subregions: MWE, the Western Mediterranean Sea (including the Ligurian Sea and the Tyrrhenian Sea); MAD, the Adriatic Sea; and MIC, the Ionian Sea, and Central Mediterranean Sea. We used the Bray–Curtis dissimilarity metric and plot it in two dimensions. Samples close together in space on the plot suggest they have a more similar type of microplastics. The colors stand for the 2D density plot of sampling gears

The concentration of microplastics in offshore waters was significantly different among vertical marine layers (*p* < 0.05). Surface water samples had a mean abundance of 0.027 microplastics · m^–2^ (number of MPs = 1567), while subsurface samples had a mean abundance of 0.007 microplastics · m^–2^ (number of MPs = 442). In addition to concentration, microplastics differed in shape among vertical marine layers. The nMDS plot revealed that although surface samples taken with manta nets (both on the starboard and port sides) are characterized by a similar proportion of MP types (e.g., fragments, sheets, filaments, and fibers), subsurface samples had a predominance of fibers (Fig. 5).

## Discussion

Microplastics have been found in seawaters in several recent studies, and when compared using the same unit of measurement (items·m^–2^) and mesh size of the tools (330 µm), our findings are consistent with those of others, such as those found in Tuscany’s coastal waters (0.069 ± 0.083 items·m^–2^; Baini et al. 2018), in the Ligurian and Tyrrhenian Seas (0.25 ± 0.84 items·m^–2^; Caldwell et al. 2020), and both in the Ionian and Tyrrhenian coasts (0.13 ± 0.19 items·m^–2^; Marrone et al. 2021). As observed in the Mediterranean seawaters (Simon-Sánchez et al. 2022), the results appear to reflect the high variability found in all three Italian subregions, with coastal mean values of 0.0616 items m^–2^ as compared to 0.0207 items m^–2^ in offshore waters.

In line with most of the published studies (Barrows et al. 2018; Goldstein et al. 2013; Llorca et al. 2020; Suaria et al. 2016), shape and colors of MPs are highly heterogenous in all three sampling areas. The study confirms the predominance of fragments blue or black in seawater surface, as still reported by some authors (Alomar et al. 2016; Pini et al. 2018; Suaria et al. 2016; de Lucia et al. 2018; Güven et al. 2017).

Consistently with previous studies (Atwood et al. 2019; Coll et al. 2012; Desforges et al. 2014; Pini et al. 2018), the concentration of MPs in the Ligurian and the Adriatic Seas decrease with distance from the coast. Sampling sites close to the coast show significantly higher MP concentrations than offshore waters (12–24 NM).

On the contrary, results from the Ionian Sea are not significantly affected by coastal distance. In this area (Gulf of Taranto), the mapping of the mesoscale and large-scale geostrophic circulation shows the presence of an anticyclonic gyre occupying the central open sea (Pinardi et al. 2015). Sea currents could influence the accumulation and transport of MPs (Liubartseva et al. 2018; Mansui et al. 2020; Zhang 2017), and the generation of eddies in the Gulf of Taranto could alter the flow of microplastics, hiding the gradient found in other sampled areas.

The concentration and distribution of MPs in the water surface are highly variable due to seasonal changes in river outflows, currents, mechanisms of degradation and fragmentation, changes in litter size, shape, buoyancy, and movement to and from other compartments (Atwood et al. 2019; Cózar et al. 2015; GESAMP 2019; Jambeck et al. 2015; Mansui et al. 2020). The abundance of MPs can be influenced by processes operating over hours, days, weeks, or months; including tidal conditions, short-term wind and rain events, and seasonal extremes (GESAMP 2019). In coastal areas these phenomena are stronger and more variable than in the open sea (Hamid et al. 2018). Nevertheless, the coastal samples were collected in autumn and spring, and we found no adverse weather conditions or season differences in microplastic concentration. Integrating environmental data would be very beneficial for accurately understanding the transport and accumulation of plastic (such a re-elaborations of rainfall data obtained at various periods). Additionally, a recent study suggested that rainfalls could release droplets from the surface water layer and thus transport microplastics into the atmosphere (Lehmann et al. 2021). Marine litter inputs come mainly from land-based sources, where anthropogenic pressures persist locally (Jambeck et al. 2015), and disperse and decrease widely in the open sea (Gorman et al. 2020). Decreasing abundance from inshore sample to offshore sample could be due to a dilution process from the input point, and MPs sink to the bottom.

In the offshore sampling waters, we made a comparison between surface and subsurface marine layers. Our results show that a significant difference occurs in the abundance of MPs collected in the two layers. The surface samples had a greater concentration of microplastics compared to the subsurface waters (0.027 microplastics m^–2^ vs 0.007 microplastics m^–2^, respectively), suggesting that MPs decrease drastically with depth, as also highlighted by Kooi et al. (2016) and Reisser et al. (2015). Furthermore, the two marine water layers are characterized by the predominance of different MP types. The nMDS analysis highlights a higher abundance of fibers in subsurface samples, suggesting a greater sinking capacity of these particles. Despite the use of two different sampling methods which may adversely impact the comparability of data, our results closely align with those found by other authors. Indeed, at the surface, fragments from plastic products were often numerically dominant, followed by plastic spheres, due to their high buoyancy (Chubarenko et al. 2016). Differently, filaments and fibers are the most important source of plastic pollution on the seabed. This could be due to the high density of different polymers used in textile manufacturing (such as polyesters; Woodall et al. 2014). Moreover, fibers are characterized by a high surface-area-to-volume ratio, which expose these particles to biofouling resulting in reduced buoyancy (Fischer et al. 2022).

In conclusion, sharing data on microplastics in seawaters may improve understanding of marine litter, reduce bias, increase future research, and facilitate the development of a global action plan. GESAMP (2019) emphasizes the importance of conducting an initial survey in order to establish a baseline, which is required in order to track future changes in the type, abundance, and distribution of plastic marine litter. This study found that microplastics are more prevalent along coasts than in open water since most plastic pollution in the sea originates from beaches, riversides, and inland areas (Auta et al. 2017; Campanale et al. 2020; Jambeck et al. 2015). In light of this result, it becomes clear how important it is to plan remediation actions and programs to minimize microplastic accumulations in the sea (e.g., efficient waste management). In addition, the fate of microplastics in the water column is affected by the sinking dynamic of these particles. Our results indicate an exponential decrease in concentration and type of microplastics from the surface to the subsurface layers. In the view of monitoring, this result is particularly important because it highlights the need for further research to better understand the dispersion of microplastics from the surface to the deep sea. As planktonic feeders consume significantly more microplastics than other commonly studied taxa (Covernton et al. 2021), and given the link between ingested microplastics and environmental plastics (Alomar et al. 2020; Franceschini et al. 2021; Sbrana et al 2020; Sbrana et al 2022), it is essential to understand the mechanisms underlying MPs aggregation in seawaters. This will help to predict and evaluate the effects of MPs contamination on marine organisms.

## Data Availability

The datasets used and/or analyzed during the current study are available from the corresponding author on reasonable request.
